# Surface Iodide Defects Control the Kinetics of the CsPbI_3_ Perovskite Phase Transformation

**DOI:** 10.1021/acsenergylett.4c01465

**Published:** 2024-08-15

**Authors:** Zachery R. Wylie, Mirella Al Katrib, Rory Campagna, Jonathan E. Outen, Samuel Smith, Peter Ruffolo, Baptiste Bérenguier, Muriel Bouttemy, Philip Schulz, Jeffrey A. Christians

**Affiliations:** † Department of Engineering, Hope College, Holland, Michigan 49423, United States; ‡ Department of Chemical Engineering, University of Washington, Seattle, Washington 98195, United States; § IPVF, Institut Photovoltaïque d‘Île-de-France, 18 Boulevard Thomas Gobert, Palaiseau 91120, France; ∥ Institut Lavoisier de Versailles (ILV), Université de Versailles Saint-Quentin-en-Yvelines, Université Paris-Saclay, CNRS, UMR 8180, 45 Avenue des États Unis, Versailles 78000, France; ⊥ Institut Photovoltaïque d‘Île-de-France (IPVF), UMR 9006, CNRS, Ecole Polytechnique, IP Paris, Chimie Paristech, PSL, 18 Boulevard Thomas Gobert, Palaiseau 91120, France

## Abstract

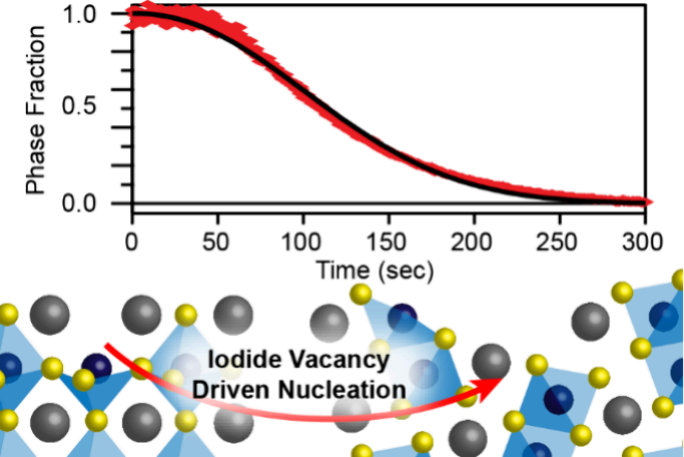

Halide perovskites are technologically interesting across a wide range of optoelectronic devices, especially photovoltaics, but material stability has proven to be challenging. One degradation mode of note is the meta stability of the perovskite phase of some material compositions. This was studied by tracking the change of CsPbI_3_ from its optoelectronically relevant perovskite phase to its thermodynamically stable nonperovskite phase, δ-CsPbI_3_. We explore kinetics as a function of surface chemistry and observe a quantitatively similar, ∼5-fold, reduction in the phase transition rate between neat films and those treated with CsI and CdI_2_. Using XPS to explore surface chemistry changes across samples, we link the reduction in the phase transition rate to the surface iodide concentration. When informed by previous theoretical studies, these experiments point to surface iodide vacancies as the nucleation sites for δ-CsPbI_3_ growth and show that phase nucleation is the rate limiting step in δ-CsPbI_3_ formation for CsPbI_3_ perovskite thin films.

Metal halide perovskites will likely grow to be disruptive materials in the world of optoelectronics. Their exceptional charge carrier mobilities,^1^ and high photoluminescent quantum yields (PLQY),^2^ make for solar cells with efficiencies as high as 26.1%.^3,4^ Coupled with low processing costs,^5^ and the possibility for their integration into tandem architectures,^6^ these solar cells promise to be transformative in the energy sector. CsPbX_3_ (X = Cl, Br, I) are the most widely studied class of all-inorganic halide perovskites due to a high thermal stability and suitable bandgap for photovoltaic applications.^7^ CsPbX_3_ exhibits three perovskite phases: cubic (α, *Pm*3*m*), tetragonal (β, *P*4/*mbm*), and orthorhombic (γ, *Pbnm*). At room temperature, it is common for CsPbI_3_ to relax locally into the orthorhombic perovskite phase.^8,9^ There also exists a nonperovskite, orthorhombic phase (δ, *Pnma*) that does not strongly absorb solar irradiation due to a wide band gap and has poor charge transport.^10^ Unfortunately, at room temperature the δ-phase is thermodynamically favored.^11^ It is known that moisture catalyzes the transition from the metastable γ-CsPbI_3_ into δ-CsPbI_3_. Also, because of ion mobility, δ-CsPbI_3_ can form in mixed cation formulations such as FA_*x*_Cs_1–*x*_PbI_3_ (FA = formamidinium).^12^ Therefore, while this class of inorganic halide perovskites is promising, the phase transition to the nonperovskite δ-phase remains the most significant hurdle to commercial implementation.^5^

Such phase transitions are important beyond just CsPbI_3_. In the hybrid organic inorganic lead halide perovskite methylammonium lead iodide (MAPbI_3_), moisture intercalates into the crystal lattice, forming metal hydrates, disrupting the structure.^13,14^ More similarly, a perovskite to nonperovskite phase transition plagues formamidinium lead iodide (FAPbI_3_) and FA-based alloys,^12,15,16^ the materials that form the backbone of most of the highest performing devices,^17^ although FAPbI_3_ transforms into different nonperovskite phases.^18^ Molecular dynamic simulations suggest that surface moisture amplifies surface halide vacancies by strongly solvating halide ions at the interface.^19^ This is also seen in the moisture-assisted self-healing of halide perovskite films.^20^ Vacancies such as these locally deform the structure resulting in octahedral tilting into the nonperovskite phase.^21,22^ This previous evidence suggests that the moisture-induced phase transition of inorganic lead halide perovskites is, at its core, a function of surface halide vacancies and ion mobility more than it is a consequence of water adsorption. In this study, we build on previous experimental and theoretical work,^22−27^ to provide a more conclusive experimental picture of the role that surface halide vacancies play in the appearance of δ-CsPbI_3_ in perovskite thin films.

Surface treatment has proven to be an effective strategy for improving the moisture resistance of perovskites,^28,29^ whether using hydrophobic surface ligands such as oleylamine to limit water adsorption and passivate defects,^30^ or filling specific vacancies by treating a film with coordinating organic ligands.^31−33^ Very recently, Guo et al. performed a comprehensive theoretical investigation into the structural collapse of CsPbI_3_.^22^ From their calculations, they concluded that kinetic, rather than thermodynamic, aspects dominate the phase stability of CsPbI_3_ and that iodide vacancies provide the nucleation sites. In this work, we aim to improve the experimental evidence for or against an iodide vacancy driven mechanism and to experimentally reveal the rate limiting step in δ-CsPbI_3_ formation. To differentiate between X-site vacancies and other effects, we passivated these vacancies using both CdI_2_ and CsI treatments. We use the Johnson–Mehl–Avrami–Kolmogorov (JMAK) model to describe the transformation and extract kinetic information on surface treatments with respect to increasing both CdI_2_ and CsI concentrations, showing that each of these treatments slows the phase transformation by a similar factor of ∼5. The analogous behavior of the two treatments, in terms of both the increase in surface iodide concentration and the slowed transition, points toward iodide vacancy filling, rather than Cs- or Pb-vacancies, as the dominant mechanism slowing the δ-phase transition. Microscopy further supports nucleation as the rate limiting step of the phase change.

In this report, CsPbI_3_ thin films spin coated on FTO glass substrates are used as a representative system to track the phase transformation. Two distinct film preparation methods were used, one with a methyl acetate antisolvent,^34^ and one with a simple one-step from DMF with no antisolvent.^35^ After annealing, as-synthesized CsPbI_3_ films were treated with a CdI_2_ or CsI solution in IPA. To control the exposure of the films to constant humidity and temperature, an ad hoc flow apparatus was built (Figure 1A), which allowed for fine control over the environment (Figure S1) by bubbling nitrogen through deionized water. It should be noted that the humid air flow in the system led to increased rates due to decreased boundary layer thickness but yielded similar data to those of CsPbI_3_ films in still air (Figure S2). The temperature of the substrate was measured directly with a thermocouple. In this way, the nitrogen flow rate determined the %RH, which allowed for a high degree of control over the kinetics of the perovskite phase transition.

**Figure 1:**
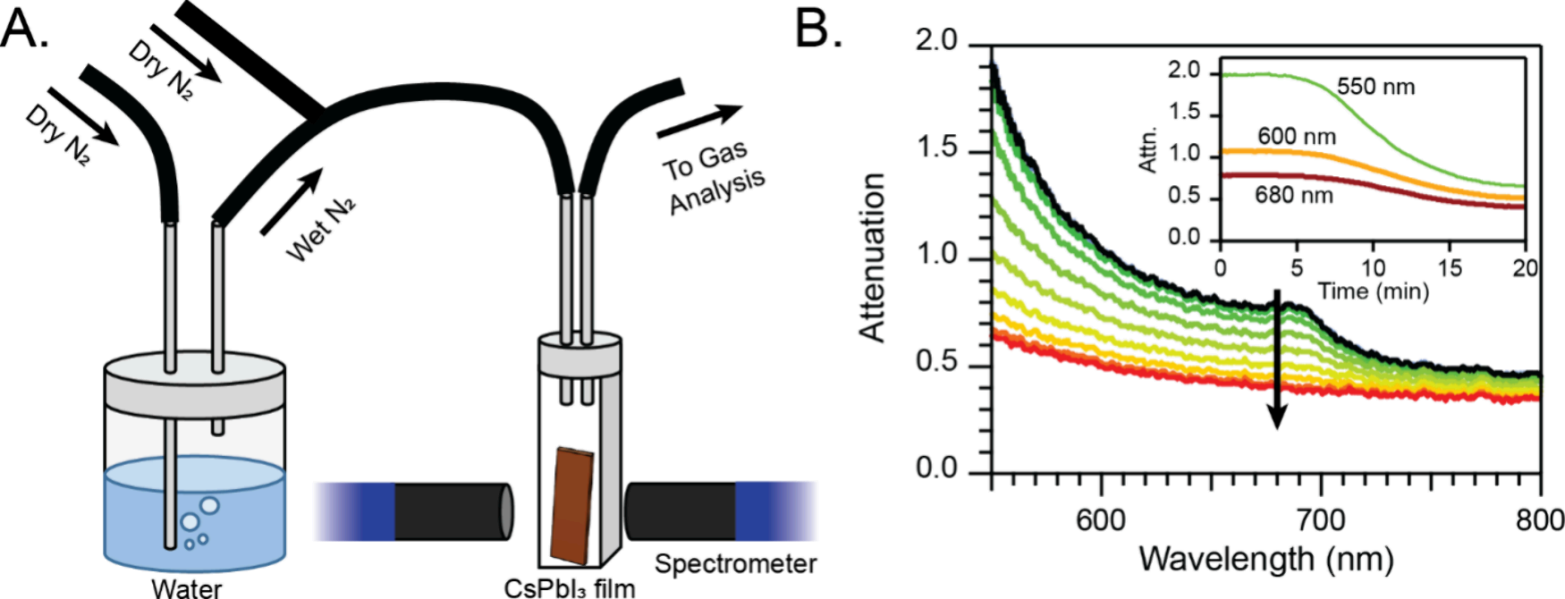
A) Diagram of the atmosphere-controlled absorption spectrometer system used for many of these experiments, and B) representative traces taken with this system as a CsPbI_3_ perovskite film undergoes a phase transition to the nonperovskite δ-phase. The inset shows kinetic traces extracted at several different wavelengths from this data.

The direct transmittance through the perovskite film was measured, and the attenuation (absorbance plus scattering/reflection) at 680 nm was used to track the phase transition over time since the black perovskite phase is easily distinguishable from the yellow nonperovskite phase at this wavelength. Figure 1B shows this phase transition as a series of attenuation measurements taken over 20 min with the black curve showing the perovskite phase at the start and the red trace showing the nonperovskite δ-phase. This spectroscopic data was then used to determine the phase fraction of the γ-CsPbI_3_ perovskite. To do this, we assume the 100% perovskite phase initially at the maximum attenuation (μ_100_) and the 100% nonperovskite phase at the minimum attenuation (μ_0_) and relate these linearly to the CsPbI_3_ perovskite phase fraction, *x* = (μ – μ_0_)/(μ_100_ – μ_0_). A typical phase transition of a CsPbI_3_ film treated only with IPA occurred over approximately 5 min at 20%RH and 20 ± 1 °C in our flow apparatus. For a clear attribution of these observed changes in the optical properties to a crystallographic phase change, we require a direct experimental probe of the structural properties. We thus confirmed by powder X-ray diffraction (XRD) measurements that the initial and final films are in fact 100% perovskite and nonperovskite phase, respectively (Figure S3). Importantly, the dynamics of the phase transition are the same as when measuring the powder XRD pattern (Figure S4) as with UV–vis spectroscopy, and the diffraction data show a quantitative agreement between the disappearance of CsPbI_3_ perovskite and the growth of δ-CsPbI_3_. The JMAK model, which is widely used to describe phase transitions in bulk and thin film systems,^36−41^ takes the following form,
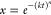
1where *k* is the effective rate constant, and *n* is the Avrami constant, or shape factor. Both *n* and *k* consist of contributions from phase nucleation and phase growth through multiple dimensions.^42^ A natural log plot of time and the natural log of the phase fraction extract the shape factor *n* as the slope of the resulting straight line as well as the natural log of the effective rate constant *k* at its intercept.
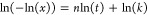
2

The JMAK model is valid for particle growth and phase transitions assuming that (i) the system is infinitely large in comparison to the size of the germinating phase, (ii) the nucleation is homogeneous throughout the material, (iii) the growth of the phase terminates when it impinges on growth from other nucleation sites, and (iv) the process is isothermal.^43−45^ The phase transition of a black CsPbI_3_ perovskite film to the yellow nonperovskite δ-phase is well described by JMAK kinetics (Figure 2 and Figure S4). However, the geometry of the system requires a more careful consideration. In thin film systems where it is possible that the size of the phase germ is no longer negligible compared to the thickness of the film, or when phase nucleation occurs at an interface, the shape factor is predicted to become nonconstant toward the end of the phase transformation when the germinated cells impinge upon an interface.^46,47^ This, however, should only be noticeable when the thickness of the film reaches an appreciable size when compared to the phase nucleation density.^48^ In the present experiments, film thickness is approximately 500 ± 100 nm (Figure S5) and is significantly smaller than the diameter of most nonperovskite domains in partially transformed films (Figure S6). However, in some experiments, a slight change in the shape factor is seen near the end of the experiments (shown in Figure 2B by the slight change in the slope as well as in some other experiments). With this in mind, the JMAK equation is appropriate for modeling CsPbI_3_ kinetics between samples if films are kept at consistent thicknesses and the temperature is constant.

**Figure 2:**
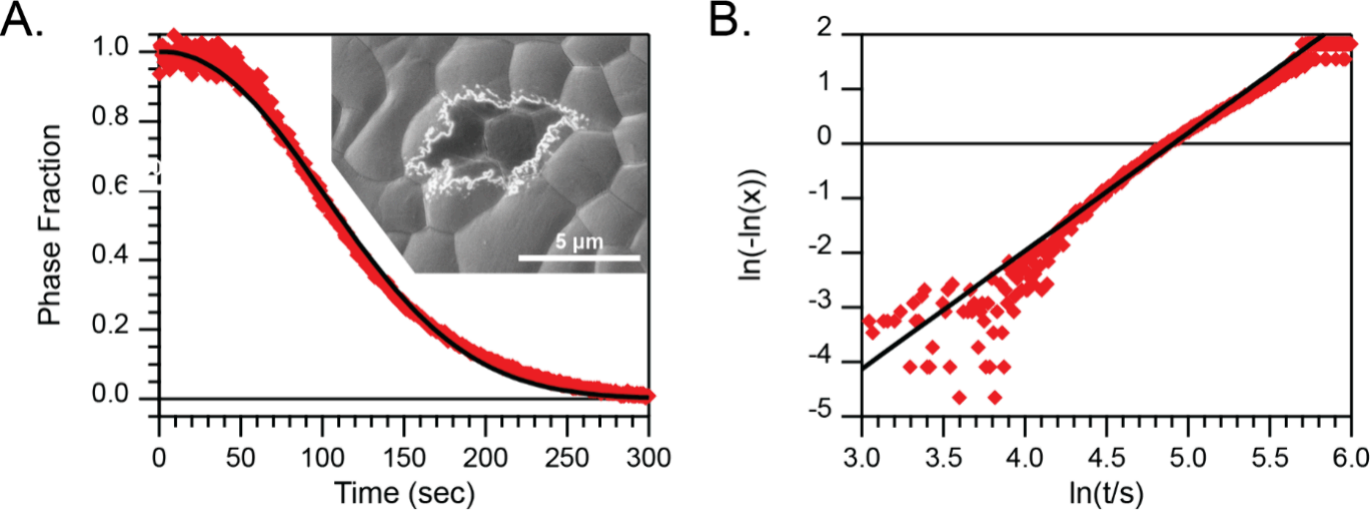
A) Transformed UV–visible spectroscopy kinetic data into phase fraction, *x*, of the CsPbI_3_ perovskite phase for a representative CsPbI_3_ thin film, fit by eq 1 (black line). The inset shows an SEM image of a central δ-CsPbI_3_ region surrounded by γ-CsPbI_3_. B) The transformation of (A) by eq 2 has a linear slope of the growth shape factor “*n*”.

Looking more closely at the representative data in Figure 2, noise is responsible for the deviation from the eq 2 model when ln(−ln(*x*)) ≥ −2 due to the asymptotic nature of the *y*-axis. The shape factor, *n*, was found to be 2.14 ± 0.29 across 10 different treated and untreated CsPbI_3_ thin films (Table S1, Figure 3). If we assume that the majority of the phase transition occurs within a 2D regime, the shape factor is expected to be 2 if nucleation of the δ-phase is homogeneous and 3 if nucleation is heterogeneous.^42,49^ Looking at SEM images of partially transformed films (see inset, Figure 2A and Figure S6), we see that the growth of the nonperovskite δ-phase is often anisotropic. Anisotropic growth would tend to reduce the shape factor. Taken together, the observed shape factor suggests that the phase transformation appears to be a mix of homogeneous and heterogeneous nucleation (from surface adsorbed water) and occurs primarily in the 2D regime. Overall, the phase transition is nucleation rate limited rather than limited by the growth rate, as clearly seen by the sparse, large crystallites in partially transformed films (Figure S6). This is similar to experiments by Lin et al. that determined a difference in these two rates of ∼10–1000 in their experiments with CsPbI_3_ microcrystals.^50^

**Figure 3:**
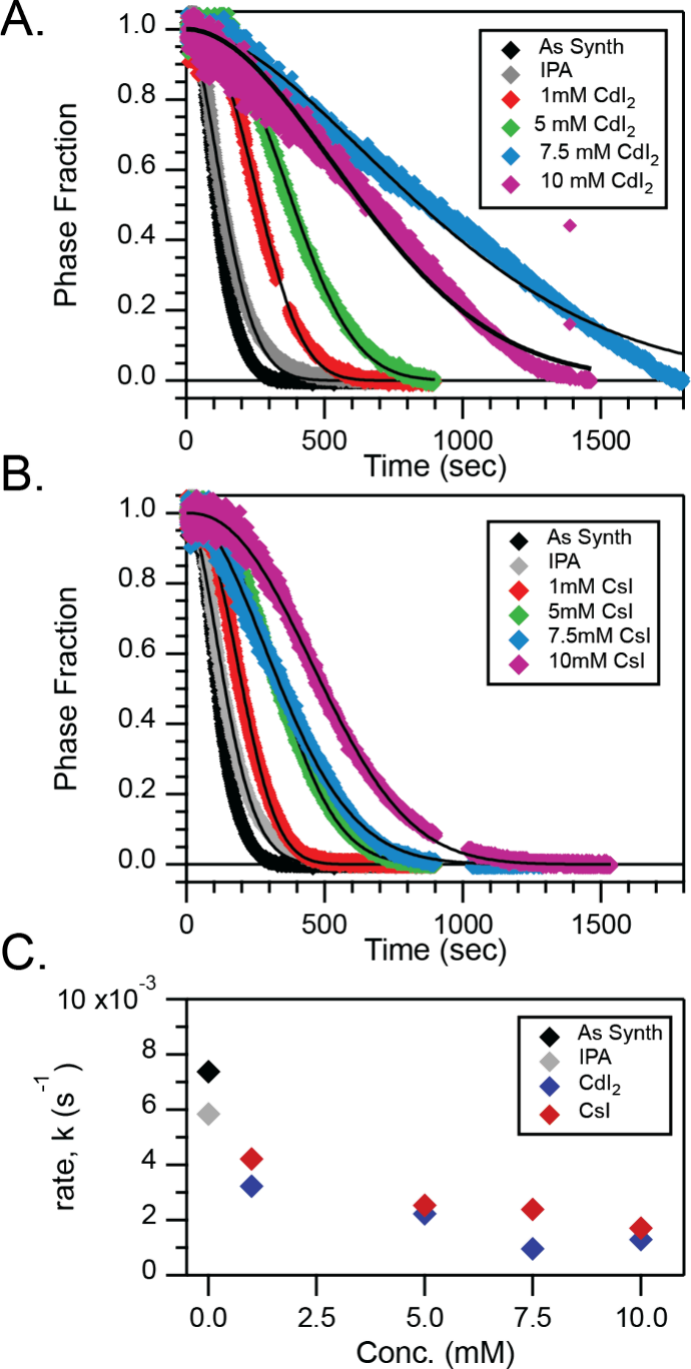
Kinetics of phase change (from UV–visible spectroscopic data) for CsPbI_3_ thin films treated with varying concentrations of (A) CdI_2_, and (B) CsI in IPA. C) The rate constant extracted from the JMAK fits (solid black lines) shows a quantitatively similar change in phase change kinetics for both treatments.

While, in the present case, the contributions of phase nucleation and phase growth are convoluted; nevertheless, these results point to the immense importance of surface chemistry in the phase transformation rate. Therefore, we selected two different surface treatments, one with CsI and one with CdI_2_, to explore their contributions to this rate. Both treatments were done following standard CsPbI_3_ film formation by coating the film with a CsI or CdI_2_ solution in IPA and annealing. These two salt treatments were selected to change the surface stoichiometry of the films to be richer in AX or BX_2_ species and thus to elucidate how these surface chemistry changes relate to the phase change kinetics.

By relating the change in attenuation at 680 nm to the phase fraction as previously described, both CsI and CdI_2_ treatments are shown to result in slower phase transition kinetics at constant temperature and humidity, despite the fact that these salts are both very soluble in water, which would make them a poor physical barrier for moisture.^51^Figure 3A,B shows plots of the phase fraction of the perovskite phase over time for different salt concentrations of CdI_2_ and CsI treatments, respectively, in a constant environment. All phase transitions, regardless of treatment, have shape factors within the range of nontreated films, which suggests that the mechanism is consistent across as-synthesized and treated films. Control films treated with pure IPA showed an effective rate constant of 5.8 × 10^–3^ s^–1^ while films treated with CdI_2_ (7.5 mM) and CsI (10 mM) showed this rate reduced approximately 5-fold to an effective rate constant of 9.6 × 10^–4^ s^–1^ and 1.7 × 10^–3^ s^–1^, respectively. As these treatments do not lead to significant changes in the initial film morphology (Figure S7), we propose that the surface chemistry is the dominant cause of the rate reduction. When the charge carrier recombination kinetics were investigated by time-resolved photoluminescence (TRPL), the treated samples showed longer lifetimes (Figure S8), consistent with a surface passivation effect. To confirm that this treatment was not limited to one specific film fabrication method, we corroborated these findings with CsPbI_3_ films made with a simple one-step process from DMF (Figure S9) as well as in multiple side-by-side trials done with films under uncontrolled ambient conditions.

Other treatments have also been found to successfully slow the CsPbI_3_ δ-phase transition, but the specific mechanism has remained somewhat obfuscated. Following a recent theoretical investigation, however, Guo et al. described iodide vacancies as, “the seed of the whole phase transition process.”^22^ Our nearly identical effects observed with CsI and CdI_2_ treatment also point to the common element, iodide, as the most likely cause behind the reduced phase transformation rate with less influence from the A- or B-site. Below, we propose a mechanism for the γ-CsPbI_3_ to δ-CsPbI_3_ phase transition based on our own observations, and informed by the literature (Figure 4). While there have been studies identifying control over transition rate through the relative stability of the γ- and δ-CsPbI_3_ phases,^52,53^ thermodynamics alone cannot account for the rate of the transition.^22^ The phase transition has been shown via DFT by Chen et al. to be a multistep process dominated by kinetics that are accelerated by iodine vacancies (*V*_I_) at the surface (Figure 4B–D) when water is introduced.^54^ These vacancies lower the barrier to the first transition state by mediating the first intermediate state with additional short-lived states that are not present in the pristine lattice phase transition pathway.^22^ Passivating *V*_I_, which in this study is done via iodine salt treatment (Figure 4A–C), raises the overall kinetic barrier and reduces the rate of δ-CsPbI_3_ nucleation. Once the δ-phase has nucleated, however, the growth proceeds rapidly in a domino effect.^55,56^ This study corroborates the conclusion that the phase transition is slowed primarily by limiting nucleation of δ-CsPbI_3_ as the shape factor from the JMAK model is similar for both the treated and untreated films, indicating that the growth mechanism is unaffected. To fully understand the effect that the CdI_2_ and CsI treatments have on the surface of the films, it is important to provide a clearer picture of the surface chemistries in treated and untreated cases to correlate the change in kinetics with *V*_I_ passivation.

**Figure 4:**
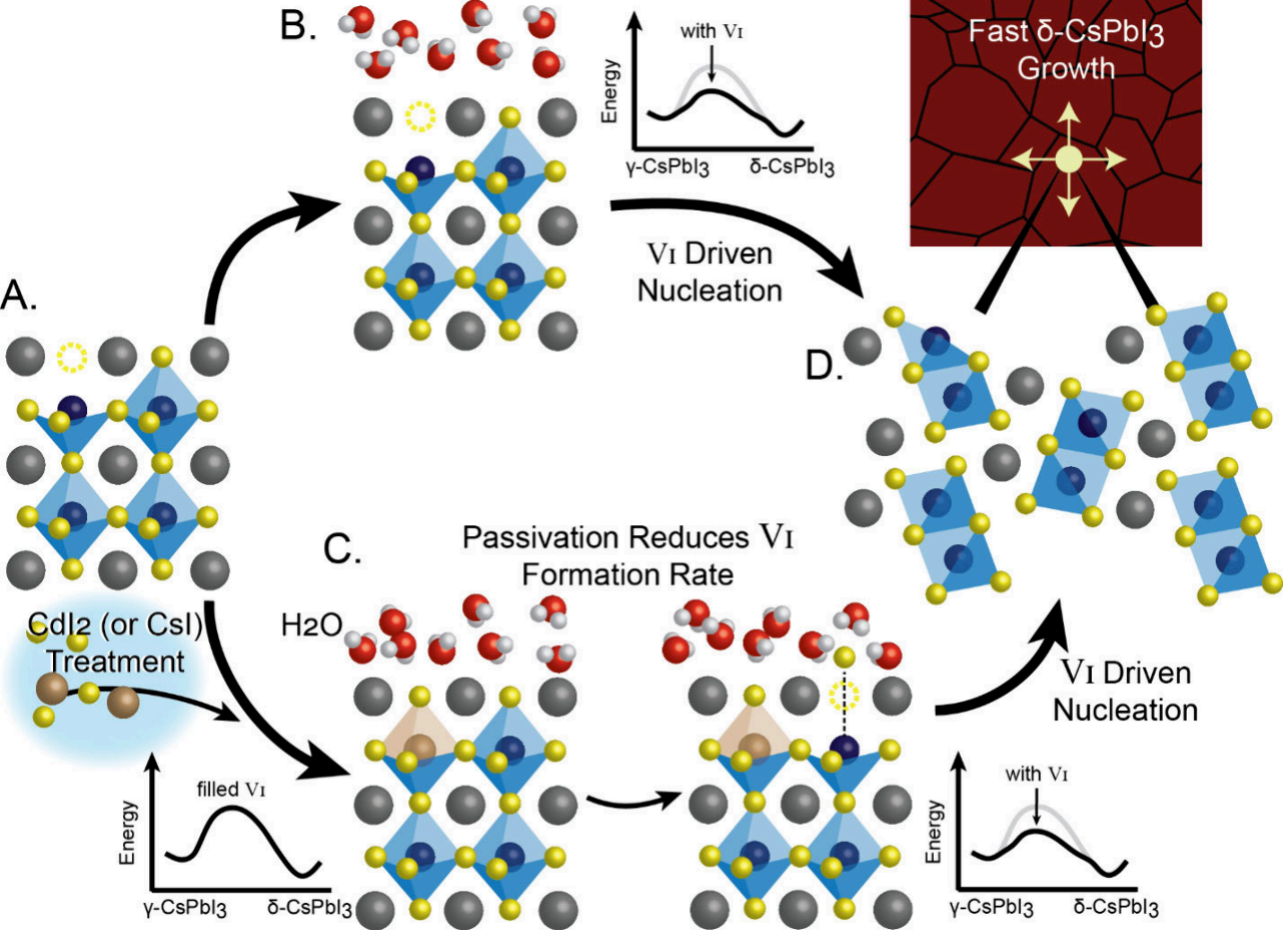
Schematic showing the proposed mechanism for the transformation from γ-CsPbI_3_ perovskite phase to nonperovskite δ-CsPbI_3_. A) As-synthesized CsPbI_3_ film with surface iodide vacancies, B) rapidly forms δ-CsPbI_3_ germs because of the reduced kinetic barrier for phase transformation caused by iodide vacancies. On the other hand, C) CdI_2_ (and CsI) treatments fill iodide vacancies, and lead to a reduced rate of δ-CsPbI_3_ germ nucleation. D) Once nucleated, the δ-CsPbI_3_ phase spreads rapidly through the perovskite film.

To accomplish this goal, X-ray photoelectron spectroscopy (XPS) measurements were performed on a range of samples, with measurements taken at two points on each sample. XPS allows for elemental quantification of the near-surface region, with the signal coming mostly from the top ∼5 nm of the sample. Thus, even though the measurement is surface-sensitive, the compositional analysis extends over the surface and the subsurface region of the probed sample. The samples included thin films of CsPbI_3_ washed with pure IPA, CsPbI_3_ treated with saturated CsI (∼10 mM) in IPA, and 4 samples of CsPbI_3_ treated with CdI_2_ at concentrations in a range from 1 mM to 10 mM in IPA. Across all of the measured samples, no visible chemical shifts in the core level spectra or rise of additional peaks stemming from a different oxidation state were observed that would indicate a change in the chemical environment of the key elements that comprise the perovskite film (Cs, I, Pb), along with trace C and O contaminations. The high energy resolution spectra for the Cs 3d, I 3d, Pb 4f, Cd 3d, and O 1s orbitals were used to quantify the surface composition (Table S3). Of the 4 samples treated with increasing CdI_2_, no specific trend was detected in surface composition of any chemical species meaning that the saturation of the surface chemical reaction is already reached for the lowest concentrations of CdI_2_ (Figure S9, Table S4). Therefore, only results for the sample treated with 10 mM CdI_2_ are presented in the main text from this sample set.

The high-resolution photopeaks of the detected elements for the samples treated with saturated CsI and 10 mM CdI_2_ in IPA can be seen in Figure 5, with references to the as-synthesized control sample. The C 1s core level is positioned at 285.2 eV and presents a shape typical for adventitious carbon contamination with effectively constant C concentration across all samples. The O 1s core level indicates the presence of oxygen contamination possibly from residual IPA, or surface contamination, which is in agreement with the C–O and C=O contributions to the C 1s spectra at higher binding energies. In addition to C–O and C=O, another oxygen species was detected at a lower binding energy of 531 eV on the treated samples. This can be attributed to the formation of an oxidized metal such as PbO_X_. However, due to the small chemical shift between Pb^2+^ and Pb^0^, combined with the low oxide proportion, no widening of the Pb 4f peaks could be identified. The emergence of a Cd 3d_5/2_ peak at 406 eV also confirms the presence of Cd at the surface (less than 1 atomic%) when treating samples with CdI_2_.

**Figure 5:**
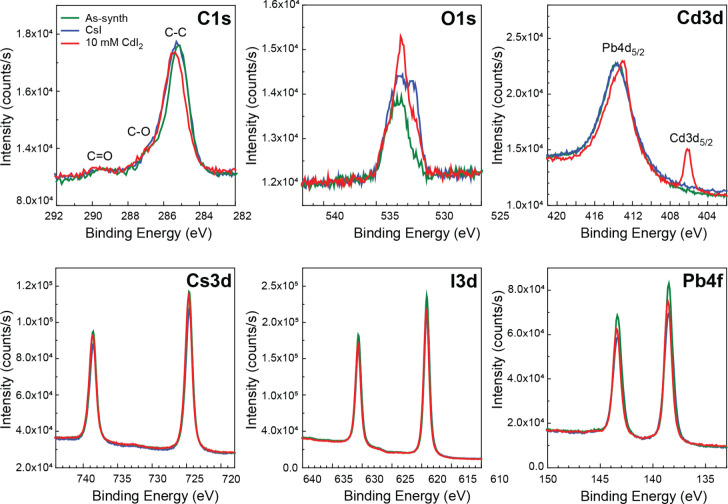
XPS measurements of the high energy resolution core levels spectra of C 1s, O 1s, Cd 3d, Cs 3d, I 3d, and Pb 4f for the untreated control film, the one treated with CsI and the one treated with CdI_2_ (10 mM).

To investigate variations in the cation and halide surface concentrations, elemental ratios were calculated for the treated and untreated samples (Figure 6). Taking the elemental ratio helps to correct for differences in surface contamination (e.g., O and C signals) and provides a better basis for sample comparison. The I/Pb ratio shows a moderate increase when the films are treated with CsI, which confirms a rise in the surface concentration of iodide after the treatment. A more significant increase in the I/Pb ratio is seen for CdI_2_ treated films; however, when the added Cd is taken into consideration for the CdI_2_-treated sample by calculating the ratio I/(Pd+Cd), we find that this new ratio is in line with the CsI treated films.^23^ In contrast, the Cs/Pb ratio presents a negligible increase compared with the sample treated with CsI and a larger increase when treated with CdI_2_. This seemingly perplexing observation that treating with CdI_2_ increases the Cs^+^ surface concentration more than a direct CsI treatment can be rationalized if Cd is filling Pb vacancies. In that case, we would expect that the CdI_2_ treated films would have a Cs/(Pb+Cd) ratio, which is in line with the neat films. In this case, the Cs/(Pb+Cd) ratio is still slightly elevated when compared to the control films and the CsI treated films, but less extreme. Despite this evidence of Cd^2+^ substitution into the CsPbI_3_ crystal, the dominant effect, from a phase stability standpoint, does appear to be the role that iodide vacancy filling plays between these two samples.

**Figure 6:**
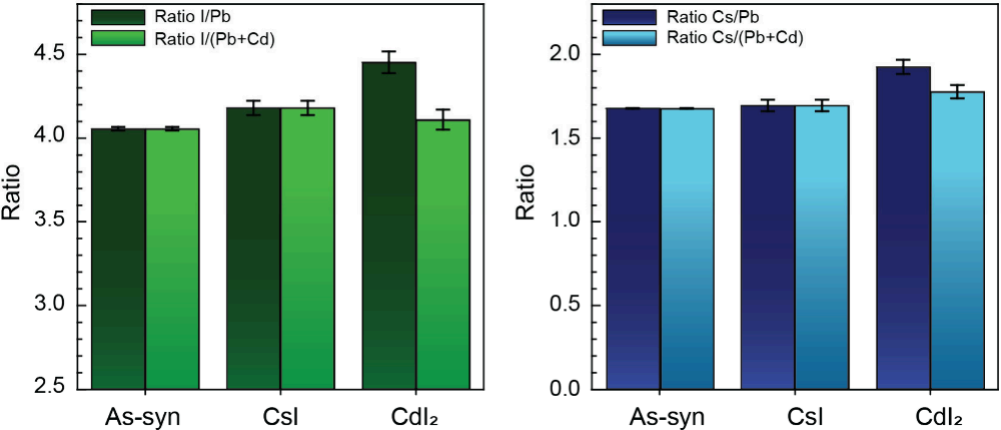
Comparison of key elemental ratios for the control film, the film treated with CsI, and the film treated with CdI_2_ (10 mM).

Controlling the phase transition from meta stable perovskite phases to nonperovskite δ-phases is critical to the stability of the majority of the halide perovskite materials that are, at present, more technologically interesting. In these experiments, we used CsPbI_3_ perovskite as a model system to explore this phase transition in greater detail. The phase transition between the perovskite phase and the nonperovskite δ-phase was found to be well-described by the JMAK model of solid-state phase transitions. Surface treatments of the CsPbI_3_ films with both CsI and CdI_2_ show quantitatively similar results; both surface treatments slow the overall rate of the phase change by approximately a factor of 5. Using XPS, we were able to confirm that both treatments result in increased surface concentrations of iodide, presumably helping to passivate or slow the formation of iodide vacancies, while the CdI_2_ treatment also appears to result in Cd^2+^ filling Pb^2+^ sites. These treatments did not change the phase transition mechanistically. More importantly, they functioned quantitatively similarly in slowing the phase transition process with similar increases in surface iodide concentrations despite differences in the concentrations of other surface species. Taken together with prior theoretical evidence, these experiments point strongly to iodide vacancies as the key nucleation sites for δ-phase formation in CsPbI_3_ perovskites. Because of their high concentrations in CsPbI_3_ thin films, we believe that the improved surface passivation of iodide vacancies will have a large kinetic effect on CsPbI_3_ perovskite phase stability. These findings, hence, denote a step toward the targeted design of halide perovskite surfaces, which enables improved control of the perovskite to nonperovskite phase transition and thus eventually the improved reliability of halide perovskite-based optoelectronic devices.

## Supporting Information Available

Experimental section, phase fraction plots, XRD spectra, SEM images, tables of kinetics parameters, tables of XPS compositions, XPS spectra (PDF)
