# Supplementary material for: Surface Iodide Defects Control the Kinetics of the CsPbI3 Perovskite Phase Transformation
Source: ACS Energy Lett. 2024 Aug 15;9(9):4378–85. doi: 10.1021/acsenergylett.4c01465 (PMC11406572; doi:10.1021/acsenergylett.4c01465)
Supplement: Supplementary file 1 — nz4c01465_si_001.pdf [file nz4c01465_si_001.pdf]

Supporting Information

Surface Iodide Defects Control the Kinetics of the $CsPbI_3$ Perovskite Phase Transformation

Zachery R. Wylie,[1,2] Mirella Al Katrib,[3,4] Rory Campagna,[1] Jonathan E. Outen,[1] Samuel Smith,[1] Peter Ruffolo,[1] Baptiste Bérenguier,[3,5] Muriel Bouttemy, [3,4] Philip Schulz,[3,5] Jeffrey A. Christians[1]*

*** Corresponding author: christians@hope.edu**

Affiliations:

1. Department of Engineering, Hope College, Holland, MI 49423, USA

2. Department of Chemical Engineering, University of Washington, Seattle, WA 98195, USA

3. IPVF, Institut Photovoltaïque d'Île-de-France, 18 Boulevard Thomas Gobert, Palaiseau 91120, France

4. Institut Lavoisier de Versailles (ILV), Université de Versailles Saint-Quentin-en-Yvelines, Université Paris-Saclay, CNRS, UMR 8180, 45 Avenue des États Unis, Versailles 78000, France

5. Institut Photovoltaïque d'Île-de-France (IPVF), UMR 9006, CNRS, Ecole Polytechnique, IP Paris, Chimie Paristech, PSL, 18 Boulevard Thomas Gobert, Palaiseau 91120, France

Experimental Methods

Materials:

Dimethyl sulfoxide (DMSO), dimethylformamide (DMF), and methyl acetate were all purchased from Sigma Aldrich. PbI_2 (99.9985%) and CsI (99.98%) were both purchased from Alfa Aesar. Cadmium iodide (99%) was purchased from Strem Chemicals. Fluorine-doped tin oxide (FTO) glass (TEC-7) was purchased from Greatcell Solar Materials. All materials were used as purchased.

$CsPbI_3$ Films and Posttreatments:

FTO glass (25 mm × 25 mm) was washed by sonication in a soap solution for 10 min followed by an additional 10 min sonication in IPA. Once completed, the slides were dried with nitrogen gas and cleaned by a 10 min UV-ozone treatment. The $CsPbI_3$ perovskite solution was created by weighing out a 1:1.05 mmol ratio of CsI to PbI_2. For each 1 mmol of CsI, 1 mL of a 4:1 v:v ratio DMF:DMSO mixed solvent was added and the solution stirred vigorously until all solids dissolved. The cleaned slides and perovskite solution were then transferred into a nitrogen glovebox. A small amount of solution (~40 μL) was spread on the FTO slides which were then spin cast at 1500 rpm for 45 seconds. With 10 sec remaining in this spin procedure, 300 μL methyl acetate antisolvent was quickly dripped onto the film. The shiny light brown films were then annealed on a hot plate for about 2.5-3.5 min at 340°C until the entire slide was dark brown. The films were removed from the hot plate and placed on the metal floor of the glovebox to cool.

$CsPbI_3$ films were formed without using this antisolvent procedure from an equimolar solution of 0.4M CsI and PbI_2 in pure DMF. This solution was spin cast at 1500 rpm for 45 seconds. The films were then annealed on a hot plate for about 5 min at 340°C. The films were removed from the hot plate and placed on the metal floor of the glovebox to cool.

CdI_2 and CsI treatments were done following one of the standard $CsPbI_3$ procedures above. A solution of CdI_2 or CsI was made in anhydrous isopropyl alcohol at concentrations ranging from 1mM to 10mM. CsI is less soluble and so, some of the more concentrated solutions were at or close to saturation, depending on temperature. To treat the $CsPbI_3$ films, the as-synthesized $CsPbI_3$ films were placed on the spin coater. About 200 μL of either CdI_2 or CsI was placed on top of the film, fully coating the film surface. After 10 s, the film was then spin cast at 1500 rpm for 45 s, and then was annealed for about 2.5-3.5 min at 340°C until the entire slide was dark brown.

Film Characterization

UV-visible absorption spectra were taken using an Ocean Insight Flame-T-TIS-VIR miniature spectrometer with OceanView software and a tungsten halogen light source. During these measurements, a portion of the film was placed inside a sealed quartz cuvette with a septum. As shown in **Figure 1a**, humid nitrogen flowed over the films to control humidity and temperature. The temperature was controlled using a JKEM Scientific Gemini temperature controller. The sampling time varied, but was typically 0.5 s per spectrum. Scanning electron micrographs were collected on a JEOL JSM-IT700HR FESEM in secondary electron imaging mode with a 2.3 kV accelerating voltage. In SEM images, the δ-$CsPbI_3$ was identified as the brighter contrast regions of the film during secondary electron imaging. This was confirmed visually using a color camera located inside the FESEM in tandem with the electron beam imaging. X-ray diffraction

measurements were taken using a Rigaku MiniFlex powder diffractometer which utilized a Cu kα radiation source. The XRD measurements were done in humid, ambient air.

Time-resolved photoluminescence (TRPL) measurements were recorded on $CsPbI_3$ films on glass substrates. The films were encapsulated with a second glass slide using a UV-curable epoxy (Ossila, Encapsulation Epoxy for Photovoltaics and OLEDs) to prevent moisture ingress during the measurements. The illumination was provided by a pulsed laser with 80 ps picosecond pulse width, a 1MHz repetition rate and at 640 nm of wavelength. The beam reaches the sample with a 45° angle, and the light spot is an ellipse with 140×200µm dimensions. The illumination fluence used was 10^{12} photons/cm^2/pulse. PL was then recorded using a vertical microscope, a silicon SPAD and a time-correlated single photon counting form.

XPS Characterization

XPS measurements were carried out under ultra-high vacuum (10^{-9} mbar). Film compositions were obtained using an XPS Thermo Scientific K-Alpha$^+$ spectrometer, equipped with a monochromated Al-Kα X-ray source for excitation at 1486.6 eV with an X-ray spot size of 400 µm. Calibration of the spectrometer was done using Cu and Au samples following the ASTM-E-902-94 standard procedure. A constant analyser energy (CAE) mode was used for the acquisition of high energy resolution spectra with a pass energy of 20 eV and an energy step size of 0.1 eV, without charge compensation. A survey spectrum was registered at the beginning and at the end of each point measurement to verify the stability of the area exposed to X-rays. Data treatment and peak fitting for XPS were performed using Thermo Scientific's Advantage™ software and library. To showcase high energy resolution core level spectra for the various elements, the C 1s peak at 285.2 eV was used as a reference.

Care was taken for sample transfer prior to XPS measurements. While in the US, samples were sealed into stainless steel tube with o-ring sealed end caps in a freshly purged nitrogen glovebox. A calcium sulfate drying agent was added to the transfer tube along with the samples to mitigate any moisture ingress effects during transfer. Then, the transfer tube was opened in a glovebox in France, and samples were mounted in a transfer vessel that can directly be plugged on the XPS spectrometer introduction chamber, preserving samples from air.

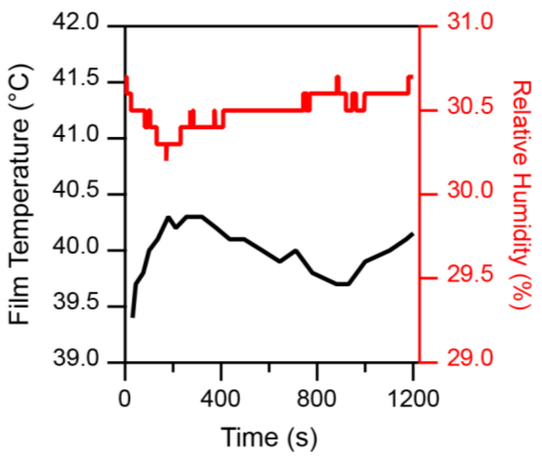

Figure S1. Representative film temperature and relative humidity taken over 20 minutes showing the stability of the experimental conditions used in this study.

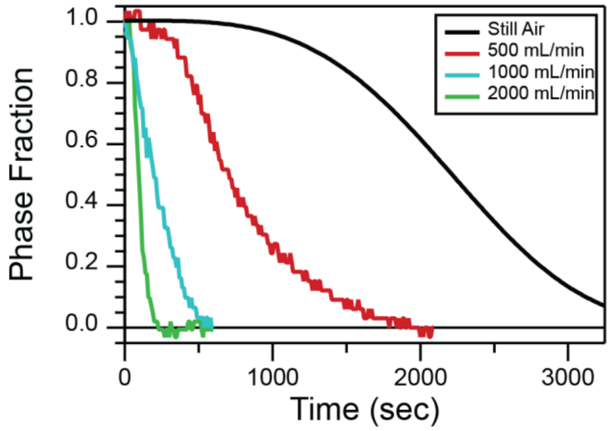

Figure S2. Phase transformation of $CsPbI_3$ from the black phase to δ-$CsPbI_3$ extracted from film absorption at 680 nm. For all experiments the film temperature was T = 20°C and the partial pressure of water in the air was measured to be P_{H_2O} = 0.70 kPa. The flow rate is the flow rate of humid nitrogen in the experiment in the flow system described herein.

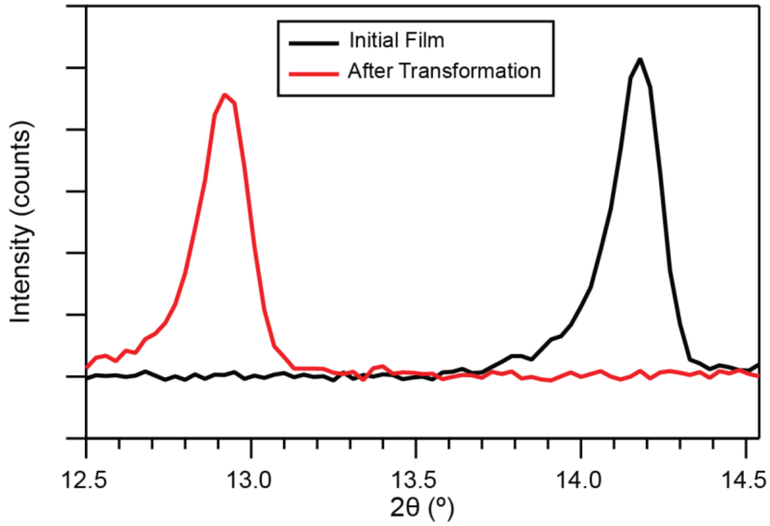

Figure S3. XRD patterns showing the primary low angle peaks of the initial, as-synthesized CsPbI3 films (black) and after this same film has gone through the phase transition to the non-perovskite phase (red).

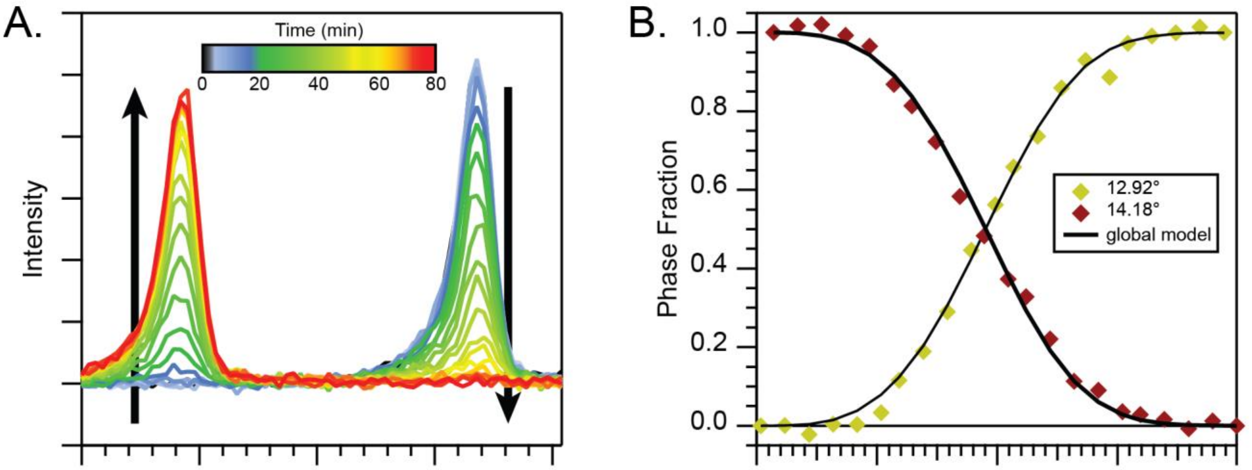

Figure S4. Tracking of the perovskite to non-perovskite phase change in a $CsPbI_3$ thin film using powder XRD. (A) Diffraction patterns showing growth of the non-perovskite phase concomitant with the reduction in the perovskite phase. (B) Kinetic analysis of this XRD data demonstrates that this transformation can be fit to the JMAK model using a single rate constant, $k = 2.36 \times 10^{-2} s^{-1}$, and growth coefficient, $n = 3.38$, and that the perovskite phase turns directly into the delta phase with no observed intermediates. Phase fraction is determined from the peak intensity.

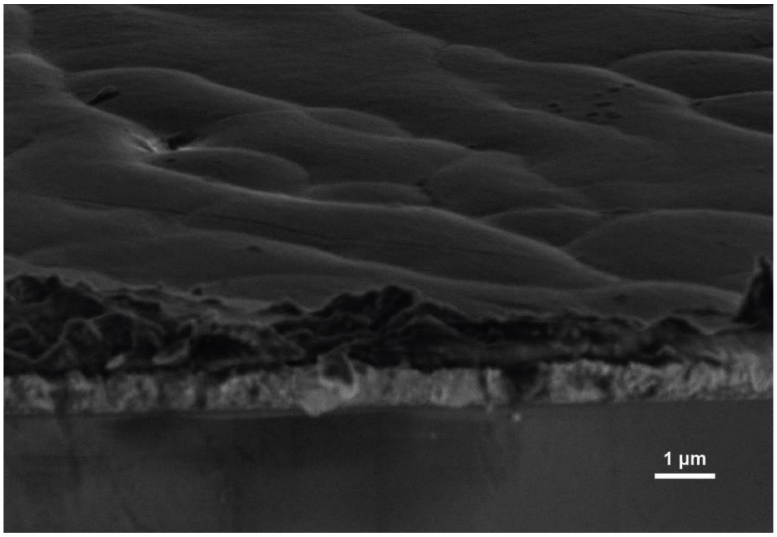

Figure S5. SEM cross section image of a $CsPbI_3$ film on an glass/FTO substrate. The image is taken at a gentle angle (10°) to show surface features as well as the cross section.

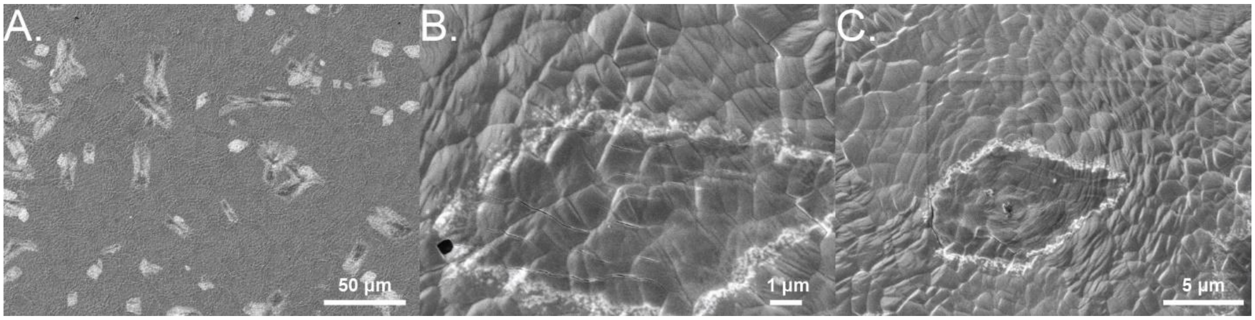

Figure S6. SEM images of a $CsPbI_3$ film that has partially undergone the phase transition. A) Lighter colored areas of δ-phase $CsPbI_3$ are scattered throughout the film. B) The δ-phase $CsPbI_3$ domains grow across multiple black-phase $CsPbI_3$ domains, and, as shown at a different location in C), sometimes grow with a small particle at the center of the δ-phase domain.

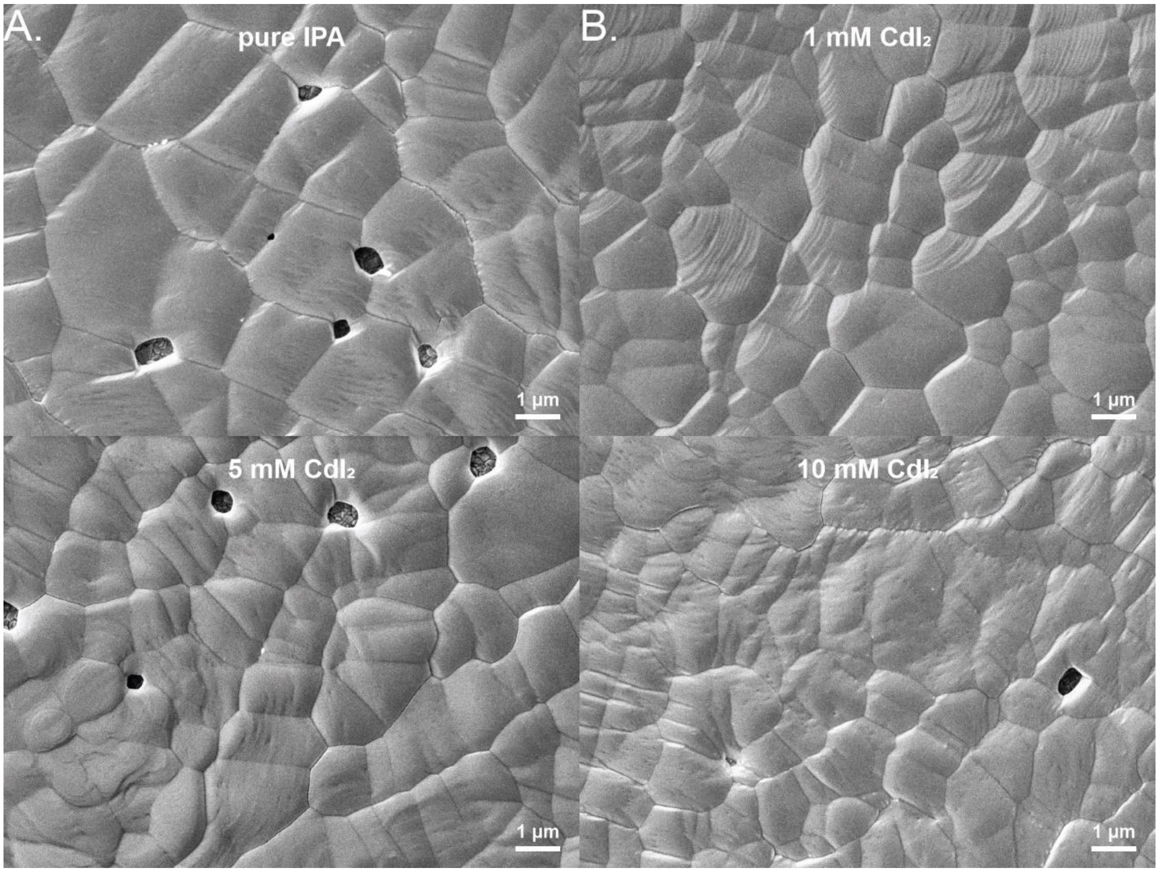

Figure S7. SEM images collected in secondary electron detection mode of $CsPbI_3$ films treated with (A) IPA, (B) 1 mM, (C) 5 mM, and (D) 10 mM CdI_2 dissolved in IPA.

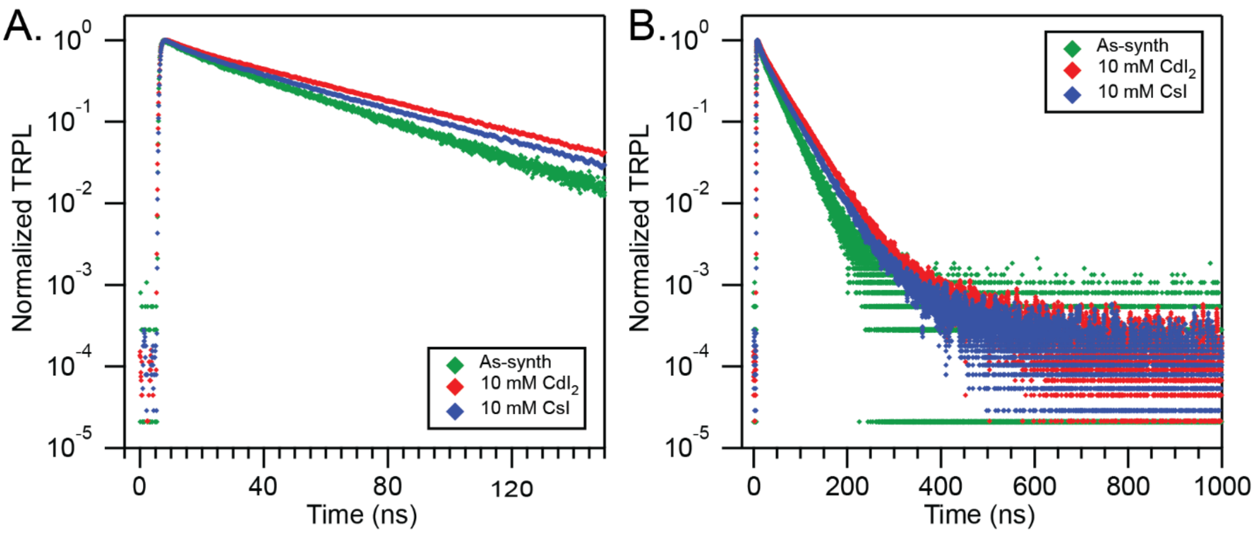

Figure S8. TRPL data for $CsPbI_3$ films fabricated with the anti-solvent method and treated with either CsI or CdI_2 at short (A) and longer (B) timescales. The decay was mono-exponential across 3 orders of magnitude for all three samples with lifetimes of approximately 34 ns (as-synth), 40 ns (CsI), and 45 ns (CdI_2).

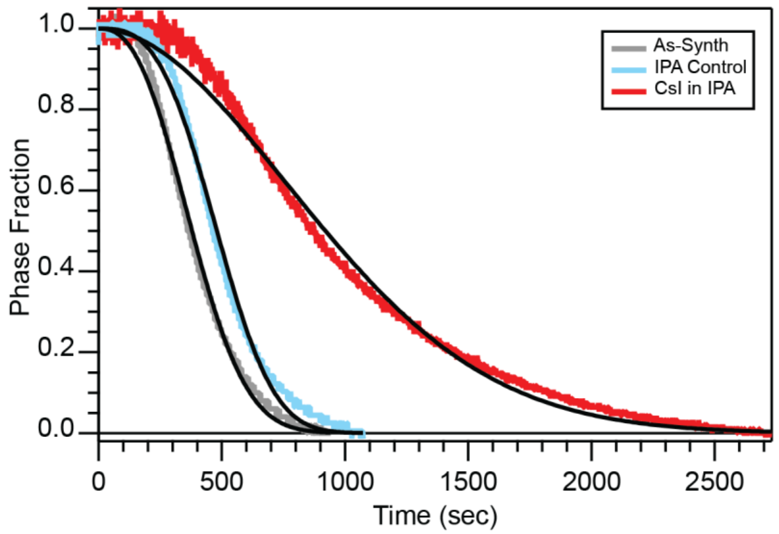

Figure S9. Comparison of the phase transformation rate in $CsPbI_3$ films made from a solution of 0.4M $CsI:PbI_2$ in DMF without an antisolvent. The CsI treatment was done with a saturated solution of CsI in IPA. For these experiments the film temperature was T = 20°C and the partial pressure of water in the air was measured to be P_{H_2O} = 0.46 kPa. The fitting parameters are shown in **Table S2**.

Table S1. Summary of the fitting parameters for the data presented in **Figure 3**. The reported error is the uncertainty in the fitting. Films were made using DMF:DMSO and MeOAc antisolvent. The column "t @ (x=0.5)" records the time when 50% of the film has transformed to δ-$CsPbI_3$.

**Film**	**t @ (x = 0.5) (s)**	**k (s^{-1})**	**n**	**χ^2**
Control	111	0.00738 ± 9.1e-6	2.165 ± 0.008	0.376
IPA	131	0.00584 ± 1.2e-5	1.790 ± 0.009	1.01
1mM CdI_2	257	0.00323 ± 5.0e-6	2.498 ± 0.01	1.24
5mM CdI_2	381	0.00223 ± 2.4e-6	2.515 ± 0.009	1.10
7.5mM CdI_2	878	0.000957 ± 1.2e-6	1.743 ± 0.006	4.39
10mM CdI_2	683	0.00129 ± 4.1e-6	1.922 ± 0.017	19.8
1mM Cs_I	197	0.00421 ± 4.1e-6	2.332 ± 0.007	0.441
5mM CsI	333	0.00253 ± 1.7e-6	2.334 ± 0.005	0.365
7.5mM CsI	341	0.00239 ± 2.9e-6	1.882 ± 0.006	1.27
10mM CsI	488	0.00171 ± 1.0e-6	2.298 ± 0.005	0.623

Table S2. Summary of the fitting parameters for the data presented in **Figure S8**. The reported error is the uncertainty in the fitting. Films were made from a single step using DMF (no antisolvent).

**Film**	**k (s^{-1})**	**n**	**χ^2**
Control	0.00228 ± 3.3e-6	2.494 ± 0.013	0.510
IPA	0.00185 ± 2.6e-6	3.027 ± 0.017	0.804
CsI	0.000899 ± 1.1e-6	1.927 ± 0.007	2.16

Table S3: Atomic composition variation of the elements detected at the surface of an untreated control film and films treated with IPA, CsI (sat., ~10 mM), and CdI_2 (10 mM).

	**As-synthesized**	**Treated with IPA**	**Treated with CsI**	**Treated with 10 mM CdI_2**
**Atomic composition (%)**				
O1s	5.6 ± 0.4	9.5 ± 0.1	7.3 ± 2.3	8.6 ± 0.3

C1s	25.5 ± 0.2	38.4 ± 11	25.2 ± 1.4	23.5 ± 0.4
Cs3d5	17.2 ± 0.1	11.7 ± 2.7	16.6 ± 1.1	17.5 ± 0.3
I3d5	41.5 ± 0.4	32.2 ±7.4	41.0 ± 2.2	40.5 ± 0.4
Pb4f7	10.2 ± 0.1	8.2 ± 1.6	9.8 ± 0.4	9.1 ± 0.0
Cd3d5	0.0 ± 0.0	0.0 ± 0.0	0.0 ± 0.0	0.76 ± 0.0
**Atomic Ratios**				
I/Pb	4.06 ± 0.01	3.93 ± 0.11	4.18 ± 0.04	4.45 ± 0.06
Cs/Pb	1.68 ± 0.00	1.43 ± 0.04	1.69 ± 0.03	1.92 ± 0.04

Note: When we treat the samples with additives dissolved in IPA, the elemental atomic percentage variations due to the presence of the solvent are different than if we treat the samples just with neat IPA since the IPA would dissolve some of the excess ions/salts from the film surface. The ratios presented for the treated samples are closer to those of the untreated control film than the ones treated with IPA.

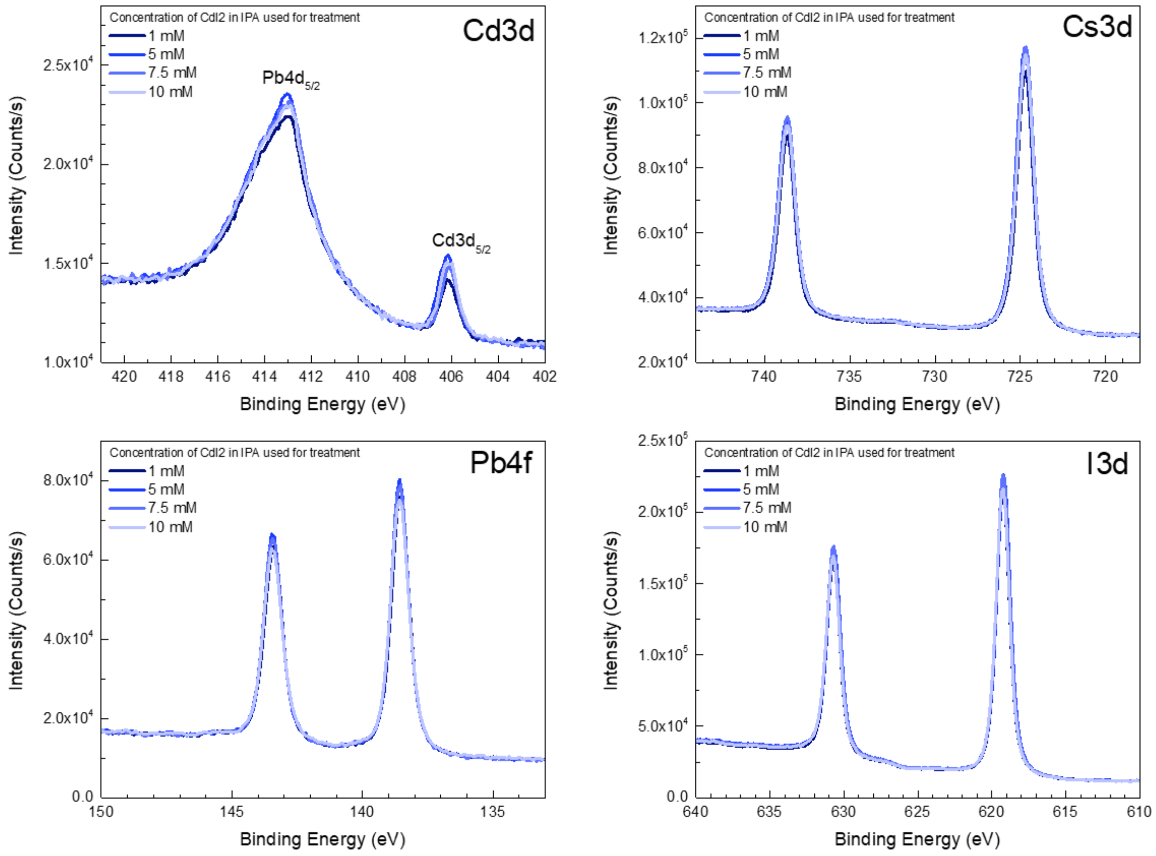

Figure S9. XPS measurements of the high energy resolution core levels spectra of Cd 3d, Cs 3d, and Pb 4f and I 3d for the films treated with CdI_2 at different concentrations.

Table S4: Atomic% variation of the elements detected at the surface of the films treated with CdI_2 at different concentrations.

	1 mM CdI_2	5 mM CdI_2	7.5 mM CdI_2	10 mM CdI_2
**Atomic composition (%)**				
O1s	5.1 ± 0.0	5.1 ± 0.5	7.5 ± 2.0	8.6 ± 0.3
C1s	29.1 ± 1.5	22.7 ± 3.3	29.4 ± 8.9	23.5 ± 0.4
Cs3d5	16.3 ± 0.4	18.5 ± 1.1	16.1 ± 3.0	17.5 ± 0.3
I3d5	39.9 ± 0.9	43.5 ± 2.2	37.9 ± 6.7	40.5 ± 0.4
Pb4f7	9.1 ± 0.2	9.4 ± 0.5	8.5 ± 1.2	9.1 ± 0.0
Cd3d5	0.51 ± 0.01	0.84 ± 0.04	0.57 ± 0.11	0.76 ± 0.02
**Atomic Ratios**				
I/Pb	4.40 ± 0.02	4.63 ± 0.01	4.45 ± 0.17	4.45 ± 0.06
Cs/Pb	1.80 ± 0.01	1.97 ± 0.02	1.89 ± 0.09	1.92 ± 0.04
Cd/Pb	0.06 ± 0.00	0.09 ± 0.00	0.07 ± 0.00	0.08 ± 0.00
